# Review of data and knowledge gaps regarding yellow fever vaccine-induced immunity and duration of protection

**DOI:** 10.1038/s41541-020-0205-6

**Published:** 2020-07-06

**Authors:** J. Erin Staples, Alan D. T. Barrett, Annelies Wilder-Smith, Joachim Hombach

**Affiliations:** 1 Arboviral Diseases Branch, U.S. Centers for Disease Control and Prevention, Fort Collins, CO USA; 2 Department of Pathology and Sealy Institute for Vaccine Sciences, University of Texas Medical Branch, Galveston, TX USA; 3 Institute of Public Health, University of Heidelberg, Heidelberg, Germany; 4 London School of Hygiene and Tropical Medicine, London, UK; 5 World Health Organization, Geneva, Switzerland

**Keywords:** Viral infection, Public health

## Abstract

Yellow fever (YF) virus is a mosquito-borne flavivirus found in Sub-Saharan Africa and tropical South America. The virus causes YF, a viral hemorrhagic fever, which can be prevented by a live-attenuated vaccine, strain 17D. Despite the vaccine being very successful at decreasing disease risk, YF is considered a re-emerging disease due to the increased numbers of cases in the last 30 years. Until 2014, the vaccine was recommended to be administered with boosters every 10 years, but in 2014 the World Health Organization recommended removal of booster doses for all except special populations. This recommendation has been questioned and there have been reports of waning antibody titers in adults over time and more recently in pediatric populations. Clearly, the potential of waning antibody titers is a very important issue that needs to be carefully evaluated. In this Perspective, we review what is known about the correlate of protection for full-dose YF vaccine, current information on waning antibody titers, and gaps in knowledge. Overall, fundamental questions exist on the durability of protective immunity induced by YF vaccine, but interpretation of studies is complicated by the use of different assays and different cut-offs to measure seroprotective immunity, and differing results among certain endemic versus non-endemic populations. Notwithstanding the above, there are few well-characterized reports of vaccine failures, which one would expect to observe potentially more with the re-emergence of a severe disease. Overall, there is a need to improve YF disease surveillance, increase primary vaccination coverage rates in at-risk populations, and expand our understanding of the mechanism of protection of YF vaccine.

## Introduction

Yellow fever (YF) virus, a mosquito-borne flavivirus, is present in tropical areas of Africa and South America. Infection in humans can produce a hemorrhagic fever and is fatal in 30–60% of persons with severe disease^1,2^. Recent decades have witnessed an unprecedented emergence of YF virus activity, including in highly urbanized areas where vaccination coverage was low^3–5^. It has been recently estimated that roughly 400 million individuals require vaccination within at-risk zones to potentially prevent epidemic of the disease though many more might be at risk due to the recent expansion of risk zones, particularly in Brazil^3,6^

YF vaccine was first developed in the 1930s after successful attenuation of the Asibi strain of YF virus to generate the strain 17D^7^. Today, three substrains (17D-204, 17DD, and 17D-213) are used as vaccines and are manufactured by six companies, of which four are prequalified by the World Health Organization (WHO)^8^. The vaccine is given as one dose either by subcutaneous or intramuscular administration, with 80% of vaccine recipients develop neutralizing antibodies 10 days post immunization and close to 100% by one month post immunization in clinical trials^9^. However, it has been noted that children <2 years of age can have lower seroconversion rates following a single dose of YF vaccine^10^. No human efficacy studies have ever been performed with the vaccine, but protection has been robustly demonstrated. Evidence for this conclusion include (1) reduction of laboratory-associated infections in vaccinated workers, (2) observation following initial use of the vaccine in Brazil and other South American countries that YF occurred only in unvaccinated persons, (3) rapid disappearance of cases during YF vaccination campaigns initiated during epidemics, (4) very few vaccine failures detected in any endemic country, and (5) protection of rhesus monkeys against virulent wild-type (WT) YF virus challenge by neutralizing antibodies generated in response to YF vaccination^11–13^.

A booster dose requirement for YF vaccine was first put into place in 1959 under the precursor to International Health Regulations (IHR), International Sanitary Regulations, with booster doses initially being required every 9 years based on available data^14,15^. The booster dose interval was changed in 1965 to every 10 years based on limited evidence from two published studies that showed neutralizing antibodies were present in most vaccine recipients, including those who received the vaccine in childhood, for at least 10 years after vaccination^16,17^. Starting in late 2011, the WHO Strategic Advisory Group of Experts (SAGE) on Immunization YF working group conducted a systematic review of ~17 unpublished and published studies that identified a very low number of vaccine failures and high seropositivity rates following vaccination over time^18,19^. From these additional, albeit observational data, SAGE concluded that a single primary dose of YF vaccine is sufficient to confer sustained immunity and lifelong protection against YF disease, and that a booster dose is not needed, except for special populations (e.g., immunocompromised and immunosuppressed)^20^. In May 2014, the World Health Assembly adopted the recommendation to remove the 10-year booster dose requirement from the IHR, which was enacted in June 2016^21^. In 2014, the United States Advisory Committee on Immunization Practices (ACIP) YF vaccine working group conducted a similar systematic review of YF vaccine immunogenicity^10^. However, since SAGE’s recommendation removed the IHR requirement for boosters, ACIP working group reviewed the available data to determine whether or not booster doses were needed as ACIP had never recommended a booster dose of the vaccine before. Based on the available data, ACIP voted in 2015 that a single primary dose of YF vaccine provides long-lasting protection and is adequate for most travelers^22^. However, as a precautionary measure, it was noted that a booster dose may be given to travelers who received their last dose of YF vaccine at least 10 years previously and who will be in a higher-risk setting based on season, location, activities, and duration of their travel. This would include travelers who plan to spend a prolonged period in endemic areas or those traveling to highly endemic areas, such as rural West Africa during peak transmission season or an area with an ongoing outbreak.

Subsequent to SAGE and ACIP recommendations that a single dose of YF vaccine is sufficient to provide lifelong protection in most individuals, several have questioned this decision^23–26^. Furthermore, several recent studies have noted waning antibody titers after vaccination and potential vaccine failures^27–34^. Below we note what is known about vaccine immunity, review the additional data that have been generated using full-dose YF vaccine since the SAGE recommendation in 2013, and discuss next steps to determine if booster doses of YF vaccine are needed.

## What constitutes YF vaccine immunity?

One of the key questions to know whether or not YF vaccine booster doses are needed is what constitutes protective vaccine immunity. The closest correlate of protection that exists for YF vaccination was established in one study of non-human primates vaccinated with YF vaccine and then challenged with virulent WT YF virus^11^. From this study, log_10_ neutralization index (LNI) of ≥0.7 was established as a potential cut-off for protective immunity with 51 (94%) of 54 surviving monkeys having a LNI ≥ 0.7. In comparison, only one (8%) of 12 monkeys who died when challenged had a LNI above 0.7. Currently, plaque reduction neutralization tests (PRNTs) are used to establish the quantitative titers of YF virus-specific antibodies as it uses less serum and is typically easier to perform. Current studies typically report either 90% PRNT (PRNT_90_), PRNT_80_, or PRNT_50_ titers. Although a PRNT_90_ titer is more specific as it reduces the likelihood of positive results due to cross-reactive neutralizing antibodies from other flaviviruses, it measures at the bottom of the S-shaped neutralization curve, which leads to less variability and can lead to false-negative results for lower virus-specific antibody titers^35^. PRNT_50_ titer are at the midpoint or more linear portion of an S-shaped curve making them higher, more variable and sensitive, but less specific. Most clinical trials for flavivirus vaccines use a PRNT_50_ assay with a titer of 1 in 10 as a correlate of protection^36–38^. However, LNI and PRNT have never been formally compared using standardized reagents to understand how they might relate. Furthermore, it is unclear if neutralizing antibodies as measured using current assays are the only correlate of protection. Our understanding of the role of cell-mediated immunity in both the initial immunologic response, as well as longer-term protection is advancing, but it also comes with the uncertainty of what might represent protective types and levels immunity that could prevent a person developing WT YF disease. However, there is general agreement that the pool of memory cells needs to be able to quickly proliferate when challenged to protect an individual as the incubation period of YF is typically short ranging from 3 to 6 days^24,39,40^.

The question of what constitutes vaccine immunologic memory is not unique to YF vaccine. Smallpox vaccine also was utilized before efficacy studies could be performed and the same questions about vaccine immunity are present for live-attenuated vaccines against vaccinia virus^41^. Although detection of antibodies is used to denote protective immunity following measles vaccination, it also has been documented that individuals lacking detectable neutralizing antibodies can develop secondary immune response with revaccination or exposure to measles virus suggesting that alternative types of immunity exist^42^.

Currently, whether or not the absence of detectable neutralizing antibodies represent an absence of protective immunity against WT YF disease is a critical knowledge gap for YF immunity. As noted above, it is also unclear what amount of antibody might be needed to protect someone against developing a symptomatic infection or viremia. Two studies have documented roughly one-third of individuals with preexisting YF virus-specific neutralizing antibodies fail to develop an anamnestic neutralizing antibody response (i.e., ≥4-fold or greater increase in neutralization titers) following a booster dose suggesting sterilizing immunity that is correlated with higher pre-vaccination titers^9,43^. If it is correct that an absence of detectable neutralizing antibodies following primary immunization or the development of an amnestic response following a booster vaccine dose means an absence of protection for YF in a primary vaccinee, one might have expected more cases of WT YF disease to be reported in children 4–10 years post-vaccination^33^. However, epidemiologic data from the recent outbreaks in Brazil indicate that very few cases of WT disease occurred in children, with a lower incidence of WT disease in children compared to adults^5,44^. Although this might be secondary to who is being exposed or differences in clinical attack rate, the recent outbreaks occurring near and in urban areas as well as the notable occurrence of cases in women tend to suggest children were likely exposed to the virus in these recent outbreaks. Finally, the development of an amnestic response might not equate to a lack of protection, particularly if the kinetics of the immunologic response is fast enough to blunt the viremia due to a WT infection.

## Vaccine failures

Since 2013, there has been several reports of vaccine failures, one in peer-reviewed literature plus epidemiologic reports issued by public health authorities^45–47^. The published study, which has been cited by others in editorials and reviews to support the need for booster doses, came out in 2014 during the ACIP deliberations and describe individuals having a history of YF vaccination who later develop WT YF disease^24,26,45^. The ACIP YF vaccine working group contacted the Brazil Ministry of Health (MOH) to verify that, as stated, 459 (55%) of 831 YF cases in Brazil from 1973 to 2008 were vaccine failures, including 27 (3%) primary vaccine failures (e.g., occurring after the first 10 days of vaccination but within the first 10 years of vaccination) and 432 (52%) secondary vaccine failures (e.g., occurring more than 10 years after vaccination potentially due to waning antibody titers)^45^. The Brazil MOH provided data to the working group noting that there were seven vaccine failures in Brazil from 1973 to 2008; five constituting primary vaccine failures, and two secondary vaccine failures occurring at 20 and 27 years post vaccination^10,45,48–50^. Unfortunately, there has never been a publication to clarify that the data were not accurate and it continues to be cited as evidence to support the need for booster doses^33^.

From data reported to the Pan American Health Organization (PAHO) during 2000–2014 and published on their website, 83 (7%) of 1164 of sylvatic YF cases reported from Bolivia, Brazil, Colombia, and Peru occurred in individuals who reported receiving YF vaccine^46^. More recently during the large outbreaks of YF in Brazil, an epidemiologic bulletin noted at least 11 cases of WT YF in individuals who were previously vaccinated and several more cases have been noted during a recent meeting^47,51^. Unfortunately, the information about these additional cases is very limited. It is unknown if these cases represent primary or secondary vaccine failures, whether and what confirmatory laboratory testing was performed, and the underlying medical history of the cases (e.g., immunosuppressed or compromised) that might have impacted their initial immunologic response to the vaccine or longer-term immunologic memory. Critically, given that YF IgM antibodies can persist for years following vaccination^52^, obtaining information about how the diagnosis of WT YF disease was made is important to interpret these results. Furthermore, it is important to note that not all individuals respond to YF vaccination; there is a median seroconversion rate of 99% (range 81–100%) in clinical trials^8^. Critically, for a state like Minas Gerais in Brazil with a population over 20 million, this means that even with 100% vaccination coverage more than 200,000 individuals who were vaccinated would fail to develop an immune response to the vaccine and would be at risk for developing disease if exposed.

## Seropositivity in vaccinated individuals

Since the SAGE recommendations in 2013, a number of articles have been published related to the immune response seen following YF vaccine, including cohorts of individuals in endemic and non-endemic locations, of different ages, and at different time points following vaccination. All studies used PRNT or microneutralization test for the detection of neutralizing antibodies against YF virus. However, the percent plaque reduction cut-off used and the definition of seropositivity or protection varied by study such that quantity of neutralizing antibodies measured in different studies are difficult to compare^35^. Furthermore, several of the studies did not use the international standard making comparison of seropositivity or antibody concentrations between studies further challenging^53^. The findings of these studies are summarized below.

### Humoral immunity in adults

There are data on longer-term humoral immunity for at least eight distinct cohorts of adults in both YF endemic and non-endemic areas of the world who received a full dose of YF vaccine (Table 1)^27,28,31,32,54–57^. Notably, there were no apparent differences between studies undertaken in endemic and non-endemic countries. In the first 5 years post-vaccination, seropositivity in the cohorts was >90%. At ≥10 years post-vaccination, the rates of seropositivity were generally lower ranging from 67% to 88% using PRNT_50_–PRNT_90_, except for a small cohort of healthy volunteers in the Netherlands where 97% (34/35) of individuals vaccinated with a full-dose of the vaccine were seropositive at 10 years when measured with PRNT_80_^57^. Interestingly, several of the studies saw higher rates of seropositivity 30–35 years post-vaccination compared to rates at 10–20 years post vaccination^54,56^. However, the number of individuals in the later vaccination time points are quite limited and they likely received an older vaccination formulation, which have differing quantities of vaccine virus^8^, impacting the generalizability of these results. Several other factors likely impact the overall rates of seropositivity in these studies, such as (1) proof of vaccination^27^, (2) different seropositivity cut-offs^28,32,35^, (3) different individuals at each time point post-vaccination often with different demographic (e.g., age of vaccination)^27,28,30,56^, (4) potential natural boosting for residents and travelers to endemic areas, and (5) potentially receiving an additional doses of YF vaccine^31^.

**Table 1:** Seropositivity following full-dose yellow fever (YF) vaccination in adults.

Study vaccine	Population	Type	Test and seropositivity criteria	Time post vaccination	Seropositive	
					No.	(%)
Kareko 2019^27^ 17D-204 (USA)	Non-endemic	Obs	PRNT_90_ Titer ≥ 10	1m–61y *0–3y*^*a*^ *3–12y* >*12y*	71/92 *12/13* *28/37* *22/32*	(77) (92) (76) (67)
Wieten 2016^b ^^54^ 17D-204 (Europe)	Non-endemic	Obs	PRNT_80_ IU/mL ≥ 0.5	11–40y *35–40y*^*a*^	89/99 *6/6*	(90) (100)
Collaborative Group 2014^c ^^28^ 17DD (Brazil)	Endemic	Obs	PRNT_50_ mIU/mL ≥ 794	1m–12+y *1–4y*^*a*^ *5–9y* *10–11y* ≥*12y*	561/651 *107/114* *69/83* *105/138* *163/191*	(86) (94) (83) (76) (85)
Campi-Azevedo 2019^30^ 17DD (Brazil)	Endemic	Obs	Micro-PRNT_50_ Titer > 50	1m–10+y 1m 1–5y 6–9y ≥10y	155/178 48/50 38/40 30/33 39/55	(87) (96) (95) (90) (71)
Miyaji 2017^d^^31^ 17DD (Brazil)	Endemic	Obs	Micro-PRNT_50_ mIU/mL ≥ 794	≤10y >10y	64/70 21/24	(91) (88)
Lindsey 2018^56^ 17D-204 (USA)	Non-endemic	Obs	PRNT_90_ Titer > 10	1m–53y *0–4y*^*a*^ *5–9y* *10–19y* *20–29y* ≥*30y*	200/221 *128/136* *18/19* *34/42* *13/16* *7/8*	(90) (94) (95) (81) (81) (88)
Martins 2018^e^^32^ 17DD (Brazil)	Non-endemic region of Brazil	Obs	PRNT_50_ mIU/mL ≥ 2.7 log_10_	1m 10m 8y	128/131 115/117 56/68^f^	(97) (98) (82)
Roukens 2018^57^ 17D-204 (France)	Non-endemic	Obs	PRNT_80_ Titer ≥ 10	10y	34/35	(97)

*Obs* observational, *PRNT* plaque reduction neutralization test, *IU* international units, *m* months, *y* years.

^a^Data presented in italic are subsets of total.

^b^Wieten et al^68^. also published data on 30 healthy and 15 immunocompromised individuals vaccinated at median 9 years (range 0–22 years) and median 7 years (range 0–18 years) previously, respectively; it is not known if there is overlap between the healthy population and those included in the study in the table above. Antibody titers were detected (IU/mL ≥ 0.5) in 15/15 (100%) immunocompromised and 29/30 (97%) healthy individuals.

^c^Data from vaccinees were presented in a separate paper using a lower cut-off of 2.7 mIU/mL for seropositivity. The proportion seropositive was higher for all groups: 99% seropositivity at 1–4 years post-vaccination; 88% seropositivity at 5–9 years; 86% seropositivity at 10–11 years; and 90% seropositivity at ≥12 years.

^d^Cohort contained individuals who received one dose (*n* = 59); two or more doses (*n* = 17); or unknown number of doses (*n* = 18) of YF vaccine. Data not broken down by time post-vaccination and number of doses other than to note there was no difference in the seropositivity by time post-vaccination for those receiving one dose.

^e^The 1-month and 10-month results data were obtained from original study paper by Martins et al. 2013^69^. Only includes individuals who received a full dose of the vaccine.

^f^Only includes persons who received full dose of YF vaccine and who were seropositive at 1-month and 10-month follow-up visits.

### Humoral immunity in children

There have been four additional published studies with short-term and long-term immunogenicity for children receiving a full dose of YF vaccine (Table 2). The published studies contain cohorts of children who received YF vaccination at 9–23 months of age. Of the two studies published evaluating the seroconversion rate following YF vaccination in children, the rates are highly variable within one of the studies and between the studies^58,59^. In a study of 595 children living in Colombia and Peru who received YF vaccine alone or with a tetravalent dengue vaccine on a YF vaccine backbone, the rate of seroconversion was noted to be 99.8–100% when measured by PRNT_50_ and titer ≥10^58^. These rates were similar though slightly higher than the rates seen in Mali (95–98%) among children who received a meningococcal A (Men A) vaccine either concurrently or serially with YF vaccine^59^. However, in the same Men A vaccine study, children in Ghana only achieved 68–79% seroconversion rates following YF vaccination. This same trend in lower rates of detectable antibodies between the two populations in the Men A study was seen when the cohorts were followed up at 2–6 years post-vaccination^34^. Seropositivity rates as low as 28% were reported for children in Ghana at 2.3 years post-vaccination, though the rate increased at 6 years post-vaccination to 43%, compared to 50% seropositivity among the children in Mali at 4.5 years post-vaccination^34^. When demographic (age of vaccination, sex), vaccination and exposure history (season of vaccination and pre-vaccination titers), and nutritional status were compared between the children in Mali and Ghana, no significant differences were identified to explain the different rates of seropositivity between these two populations^60^. In the second study evaluating longer-term immunity in different cohorts of children in Brazil up to 10 years post-vaccination, a substantial decline was noted in the seropositivity rates over time^33^. Using a titer ≥10 with PRNT_50_, 54% of children were not seropositive at 7 years post-vaccination. Although the rates of seropositivity increased when using a lower titer cut-off (PRNT ≥ 5), 36% of children at 7 years post-vaccination lacked detectable neutralizing antibodies.

**Table 2:** Seropositivity following full-dose yellow fever (YF) vaccination in children.

Study Vaccine	Population	Type	Test and seropositivity criteria	Time post vaccination	Seropositive	
					No.	(%)
López 2016^58^ 17D-204 (France)	Endemic	RCT	PRNT_50_ Titer ≥10	1m	594/595	(99.8)
Chowdhury 2015^a^^59^ 17DD (Brazil)^Ghana^ 17D-213 (Russia)^Mali^	Endemic	RCT	Micro-PRNT_50_ Titer ≥8	1m	841^G^ 300^M^	(68–79)^G^ (95–98)^M^
Domingo 2019^34^ 17DD (Brazil)^Ghana^ 17D-213 (Russia)^Mali^	Endemic	RCT	Micro-PRNT_90_ ≥0.5 IU/mL^b^	2.3y 4.5y 6y	121/436^G^ 296/587^M^ 188/436^G^	(28)^G^ (50)^M^ (43)^G^
De Noronha 2019^c^^33^ 17DD (Brazil)	Endemic	Obs	Micro-PRNT_50_ Titer ≥10	0–6m 1y 2y 4y 7y 10y	143/165 107/140 97/136 72/122 57/135 58/126	(87) (76) (71) (59) (42) (46)

*G* Ghana, *M* Mali, *Obs* observational, *PRNT* plaque reduction neutralization test, *RCT* randomized control trial, *IU* international units, *m* months, *y* years.

^a^Numbers represent the number of children with immunogenicity results included in the respective countries (denominator) and proportions represent those seroconverting; 64–68% of children in Ghana (*N* = 38) and 90–98% of children in Mali (*n* = 12) were seropositive at baseline and had ≥2-fold increase in antibody titer.

^b^Data from vaccinees were also presented in the same paper using any detectable antibodies. The proportion of seropositive was higher for all groups: 39% (172/436) at 2.3 years in Ghana; 70% (409/587) seropositivity at 4.5 years in Mali; and 51% (223/436) seropositivity at 6 years in Ghana.

^c^Same data presented in Campi-Azevedo et al. (2019)^55^.

One potential explanation for the varying immune response both initially and potentially longer-term among the pediatric studies could be the age at which the children received their vaccine. Younger age groups might be expected to have a less robust initial immune response, potential immunologic interference from maternal antibodies, or more concomitant infections lead to a decreased immune response^61,62^. The cohorts in Mali, Ghana, and some of the children in the Brazil study received YF vaccine at 9 months of age. This is compared to children in Colombia and Peru who received the vaccine at 12 months of age and others in the Brazil cohort who were as old as 23 months when they were vaccinated. However, when the age of vaccination was assessed by the ACIP YF working group relative to the seroconversion rates, the analysis of results from aggregated studies found no difference in seroconversion rates when the children were vaccinated at 9 months of age compared to 12 months^10,22^.

With these new pediatric data, there are seemingly more questions than answers to the variability of the results between the pediatric cohorts. The authors of the studies and associated editorials question what contributes to the variability in results hypothesizing that it could be due to differences in immune microenvironment, vaccine substrains used, how the samples were handled, the test used, and potential difference in vaccine handling^33,61,63,64^. Furthermore, in both Ghana and Brazil, the authors questioned whether or not children had received another dose of the vaccine as the proportion seropositive was higher at later time points^33,34^.

### Additional immunogenicity data

Since 2013, several studies have been published regarding cellular immunity, including CD8+, CD4+, and memory phenotypes, formed in response to YF vaccine^30,54,55,65^. However, the specific impact of alternative types of immunologic memory and their role in protecting persons against disease is not well-characterized or known.

## Next steps

The studies published since SAGE and ACIP made their recommendation that one dose of YF vaccine is sufficient to provide lifelong protection in most individuals provide additional data on YF vaccine immunity. Given the heterogeneity of results, in particular for the pediatric cohorts, further studies would be welcomed.

However, the basic questions that were debated in the discussions of both SAGE and ACIP still remain, how durable is the immunity elicited by YF vaccine and what constitutes protective immunity against YF virus infection and disease? To truly address these questions, additional research and data are needed. Increased transparency and sharing of information on potential vaccine failures are critical to better understand of the >800 million doses the vaccine that have been administered how many might have failed to provide both short-term and long-term protective immunity. With this is the need to continue improving and strengthening YF disease surveillance and laboratory testing^66^, not only to detect possible vaccine failures but also to obtain samples early enough to make a definitive diagnosis of WT disease by molecular testing. In addition, every effort must be made to ascertain the vaccination status of the patient. As noted above, using standards and evaluating the correlation between neutralization titers determined by LNI and PRNT would improve our ability to compare studies and begin to set thresholds as to what antibodies levels are needed to potentially prevent WT disease. Furthermore, additional research is needed to determine the kinetics of the immune response when a vaccinee receives a booster vaccine dose or has a WT infection (e.g., does an amnestic response mean a lack of adequate protection?) and to validate the immune correlate of protection following YF vaccination using more modern knowledge and techniques (e.g., assessing the role of cellular immunity). WHO currently plans to receive input from subject matter experts on how best to proceed with measuring YF vaccine immunity in a consistent manner to allow for comparability between studies.

Overall, we expect the debate of whether or not to give booster doses of YF vaccine to continue in lieu of more data. However, one clear public health action that can and should be taken now is to improve YF vaccination coverage among children living in at risk areas. Based on WHO and UNICEF estimates of vaccine coverage (WUENIC), YF vaccination rates among children living in YF endemic areas ranges from 42% to 97% (median of 85%) in the Americas and 29–94% (median: 68%) in Africa^67^. The current large outbreaks of measles throughout the world, including in YF endemic areas where the vaccines are often given at the same visit, reinforces poor YF vaccination rates that exist among children. If children do not even receive their first dose of YF vaccine, it is hard to focus on whether they might need a booster dose. We encourage researchers, clinicians, and public health officials to continue to evaluate and publish quality data on YF vaccine immunity and vaccine failures to inform public health policy related to YF vaccine use and optimize our ability to prevent YF.
